# Frailty and cardiovascular disease: a bidirectional relationship with clinical implications

**DOI:** 10.3389/fcvm.2025.1684701

**Published:** 2025-11-07

**Authors:** Neil Johnson, Junru Qu, Kenji Wagatsuma, Yingying Su, Beibei Du, Yuquan He, Ping Yang

**Affiliations:** 1 Department of Cardiology, China-Japan Union Hospital of Jilin University, Changchun, China; 2 Tsukuba Heart Center, Tsukuba Memorial Hospital, Tsukuba, Ibaraki, Japan; 3 Changchun University of Chinese Medicine, Changchun, China

**Keywords:** frailty, cardiovascular disease, aging, risk stratification, personalized medicine, inflammation, geriatric cardiology

## Abstract

**Background:**

Frailty and cardiovascular disease (CVD) are increasingly recognized as interconnected conditions that significantly impact aging populations. This review synthesizes evidence from studies published between 2000 and 2025, identified through Google Scholar and PubMed using keywords such as “frailty”, “CVD”, “frailty assessment”, and “multicomponent interventions”. Frailty, characterized by reduced physiological resilience and increased vulnerability to stressors, affects 10%–15% of community-dwelling older adults and is associated with adverse CVD outcomes.

**Main body:**

Our analysis demonstrates that frailty and CVD share common pathophysiological mechanisms, including chronic inflammation (“inflammaging”), mitochondrial dysfunction, and endothelial impairment. The reviewed literature reveals frailty prevalence varies substantially by CVD subtype, ranging from 30% in patients with coronary artery disease (CAD) to 80% in those with heart failure (HF). Frailty independently predicts adverse outcomes, conferring a 2.5–3.5-fold higher mortality risk. While multiple assessment tools exist (e.g., Fried Phenotype, Clinical Frailty Scale), this review highlights the absence of a gold standard assessment tool for cardiovascular populations. A critical challenge is that traditional cardiovascular risk scores often fail to account for frailty, leading to significant treatment disparities. Effective management requires a paradigm shift towards multimodal interventions. Evidence supports combined exercise and nutritional programs (e.g., VIVIFRAIL, SPRINT-T), which improve physical function and frailty severity. Recent guidelines now recommend such rehabilitation. Emerging therapeutic strategies—including senolytics (e.g., dasatinib plus quercetin), stem cell mobilization, and angiogenic gene therapy—show promise for targeting shared biological pathways of vascular decline.

**Conclusion:**

The synthesis of recent evidence underscores the necessity of routine frailty assessment in cardiovascular care. Integrating validated frailty measures can improve risk stratification and enable personalized treatment. Future research should focus on standardizing assessment in cardiology and developing targeted interventions for shared pathways. Addressing frailty as a modifiable risk factor could significantly improve outcomes for older adults with CVD.

## 1 Background

As the global population ages, the intersection of frailty and CVD has emerged as a crucial area in clinical practice and research. Frailty, characterized by diminished physiological reserve and heightened vulnerability to stressors, affects 10%–15% of community-dwelling older adults and up to 50% in hospitalized settings. It elevates risks of institutionalization, caregiver burden, and mortality, particularly in patients with CVD (1).

To ensure conceptual clarity, it is important to distinguish frailty from related but distinct terms often used in the context of aging. Frailty is a distinct clinical syndrome characterized by a multisystem decline in physiological reserve, leading to increased vulnerability to stressors. It is operationally defined by criteria such as Fried's phenotypic model (e.g., unintentional weight loss, exhaustion, low activity, slowness, and weakness) or Rockwood's deficit accumulation index (2). Sarcopenia, the age-related loss of muscle mass and strength (3), is a key component and driver of physical frailty but does not encompass its full multisystem nature. While sarcopenia contributes significantly to frailty, not all frail individuals have sarcopenia, and not all with sarcopenia meet the criteria for frailty. Disability, on the other hand, refers to the difficulty or dependence in carrying out essential activities of daily living (ADLs); it is a common and serious outcome of progressive frailty but represents a separate construct. Lastly, comorbidity denotes the co-existence of multiple chronic medical conditions (e.g., diabetes, chronic kidney disease). While comorbidities increase the risk of developing frailty, they are not synonymous with it; frailty represents an overarching state of vulnerability that can be exacerbated by, but exists independently from, specific diseases. Understanding these distinctions is critical for accurate assessment, management, and research into the interplay between frailty and cardiovascular disease.

CVD remains the leading cause of death worldwide, with an estimated 20.5 million deaths reported in 2021 (4). Frailty and CVD share bidirectional relationships and common pathways, including chronic inflammation, endothelial dysfunction, and metabolic dysregulation. Despite this interplay, traditional CVD risk scores often overlook frailty, leading to underestimation of risk and suboptimal treatment in vulnerable patients. This review explores the bidirectional relationship between frailty and CVD, examining the underlying biological mechanisms, the prognostic significance of frailty, and its implications for clinical management. A comprehensive literature search was conducted using Google Scholar, PubMed, and other databases, with keywords such as “frailty”, “CVD”, “frailty assessment tools” and “multicomponent interventions”. Relevant studies published between 2000 and 2025 were reviewed from various journals and sources. Through this analysis, we aim to highlight how frailty contributes to the development and progression of CVD and how integrating frailty assessment into cardiovascular care can improve patient outcomes and inform targeted interventions.

## 2 Main text

### 2.1 Pathophysiological links between frailty and CVD

Frailty and CVD are interconnected conditions that engage in a detrimental, bidirectional relationship, significantly amplifying morbidity and mortality in older adults (5). This synergy is underpinned by two fundamental concepts: first, a set of shared biological mechanisms that simultaneously drive the pathophysiology of both conditions, and second, a series of bidirectional clinical pathways through which frailty worsens CVD outcomes and, conversely, CVD accelerates the progression of frailty. The following sections will detail this model, explaining how the shared substrate of aging-related decline creates a vicious cycle that is manifested in specific clinical interactions. Understanding this integrated pathophysiology is crucial for moving beyond siloed treatment and towards holistic management strategies.

#### 2.1.1 Shared biological mechanisms

Frailty and CVD converge on several fundamental biological pathways of aging. These mechanisms create a shared substrate of physiological decline, explaining their high rate of co-occurrence. Rather than operating in isolation, they form a synergistic network that accelerates multisystem dysfunction.

##### 2.1.1.1 Chronic inflammation and immune aging

A state of chronic, low-grade inflammation (“inflammaging”) is a cornerstone of both frailty and CVD, characterized by elevated levels of proinflammatory cytokines such as interleukin-6 (IL-6) and tumor necrosis factor-alpha (TNF-α). The origins of inflammaging are multifactorial, involving age-related immune dysregulation (immunosenescence), visceral adiposity, and metabolic disorders such as obesity and insulin resistance, which collectively sustain this inflammatory state (6).

In the vasculature, these cytokines are central to atherosclerotic progression. They activate pathways like the NLRP3 inflammasome—triggered by cholesterol crystals and oxidative stress to release IL-1β and IL-18—and Toll-like receptors (TLRs) that respond to oxidized LDL, collectively promoting endothelial dysfunction, monocyte recruitment, and plaque instability. The clinical validation of this pathway is demonstrated by trials showing that targeting IL-1β with canakinumab reduces cardiovascular events. However, the clinical translation of anti-inflammatory approaches remains constrained by the need to preserve essential immune functions while suppressing pathological inflammation (7). Complementing these mechanisms, dysregulated Notch and Wnt signaling alters vascular smooth muscle cell phenotype and macrophage polarization, further increasing plaque vulnerability (8).

Concurrently, in skeletal muscle, the same cytokines drive the sarcopenia central to frailty by promoting muscle protein catabolism, suppressing regeneration, and contributing to anabolic resistance. This inflammatory-mediated muscle wasting leads directly to the loss of muscle mass and strength that defines physical frailty (9).

Elevated levels of IL-6 and TNF-α show strong associations with both frailty severity and adverse cardiovascular outcomes, including myocardial infarction, HF, and mortality, underscoring their role as key mechanistic links (10, 11).

Thus, inflammaging provides a direct biological link between vascular damage and muscle wasting, and acts as a primary driver of downstream dysfunction in other key systems, including cellular mitochondria and the endothelium.

##### 2.1.1.2 Mitochondrial dysfunction and oxidative stress

Mitochondrial integrity is essential for cellular energy production and redox homeostasis. Age-related mitochondrial decline is characterized by inefficient adenosine triphosphate (ATP) production and increased generation of reactive oxygen species (ROS), creating a state of bioenergetic failure and oxidative damage (12).

Mitochondrial dysfunction critically contributes to frailty by impairing skeletal muscle energetics. Reduced ATP production diminishes muscle strength and endurance, while accumulated ROS damage proteins and accelerate proteolysis. This dual pathology - combining bioenergetic failure with oxidative damage - drives the muscle wasting and functional decline characteristic of frailty, linking cellular aging to physical vulnerability (13).

Mitochondrial dysfunction similarly drives cardiovascular pathogenesis through three interconnected mechanisms: Impaired cardiac oxidative phosphorylation (OXPHOS) reduces contractility, promoting HF; mitochondrial ROS generation damages endothelium, exacerbating atherosclerosis; and altered biogenesis via PGC-1α/TFAM, excessive Drp1/MiD49/51 -mediated fission, and defective mitophagy collectively impair energy metabolism, amplify oxidative stress, and trigger proinflammatory signaling in vascular cells. These processes collectively accelerate plaque formation and instability through lipid accumulation, endothelial impairment, and smooth muscle proliferation (14).

Compromised mitochondrial quality control results in accumulated mtDNA mutations and defective organelles, worsening cellular damage and energy depletion. Mitochondrial dysfunction thereby accelerates both cardiovascular and muscular aging. The resulting oxidative stress not only damages tissues directly but also serves as a potent stimulus for perpetuating the chronic inflammatory state and impairing endothelial function.

##### 2.1.1.3 Endothelial dysfunction

The endothelium, a key regulator of vascular homeostasis, becomes dysfunctional with age. This dysfunction is marked by reduced nitric oxide (NO) bioavailability, increased arterial stiffness, and a prothrombotic state (15, 16). In CVD, endothelial dysfunction drives atherosclerosis progression and plaque instability (17). In the context of frailty, this process contributes to decline through impaired tissue perfusion (hypoperfusion). Reduced microvascular blood flow exacerbates mitochondrial dysfunction and sarcopenia in skeletal muscle, limiting physical capacity (18). Key mediators include elevated asymmetric dimethylarginine (ADMA), while impaired Vascular Endothelial Growth Factor (VEGF)/NOS signaling further compromises NO production and endothelial progenitor cell (EPC) function reducing angiogenic capacity (16). Stiffened arteries increase cardiac afterload, while hypoperfusion exacerbates mitochondrial dysfunction, sarcopenia, and physical decline.

The state of hypoperfusion and oxidative stress resulting from endothelial dysfunction creates a tissue environment that further exacerbates inflammatory signaling and neurohormonal imbalances, linking vascular health directly to systemic physiological reserve.

##### 2.1.1.4 Neurohormonal dysregulation

Aging disrupts neurohormonal systems regulating cardiovascular and metabolic function, with both frailty and CVD involving dysfunction of the hypothalamic‒pituitary‒adrenal axis (HPA axis), the renin–angiotensin–aldosterone System (RAAS), and the autonomic nervous system (ANS).

The HPA axis contributes through cortisol-dehydroepiandrosterone (DHEA) imbalance, where an elevated cortisol-to-DHEA ratio is associated with increased frailty, immune dysfunction, and age-related diseases including osteoporosis and Alzheimer's disease (19). Elevated cortisol levels in aging promote metabolic disturbances, inflammation, and physical decline, while reduced DHEA exacerbates these effects through loss of anti-inflammatory and neuroprotective properties (20). This hormonal imbalance increases pain sensitivity, reduces muscle mass, and impairs recovery, while flattened diurnal cortisol rhythms worsen cardiovascular outcomes (21).

The RAAS critically links CVD and frailty through chronic activation that elevates angiotensin II, driving vascular stiffness, cardiac fibrosis, and endothelial dysfunction (22). Frailty exacerbates this process, as inflammatory markers such as IL-6 and C-reactive protein (CRP) further stimulate the RAAS, creating a cycle where RAAS-mediated inflammation worsens sarcopenia and frailty-related metabolic dysfunction amplifies cardiovascular damage. Clinically, this manifests as an increased risk of atrial fibrillation (AF) and poor recovery after interventions such as transcatheter aortic valve replacement (TAVR) (23, 24). At the cellular level, angiotensin II induces mitochondrial dysfunction and oxidative stress, while aldosterone excess promotes protein catabolism and insulin resistance, explaining poor outcomes in frail CVD patients (25).

Frail older adults show characteristic cortisol patterns—elevated evening levels and flattened diurnal variation—linked to muscle loss, bone deterioration, and metabolic dysfunction (26, 27). This hypercortisolemia promotes inflammation through both direct effects and reduced anti-inflammatory DHEA, while exacerbating insulin resistance and vascular damage that worsen hypertension and atherosclerosis (28).

ANS dysfunction is associated with frailty in older adults, characterized by orthostatic hypotension (OH) and impaired heart rate variability. Frail individuals show higher rates of consensus orthostatic hypotension (COH) (1.6-fold) and initial orthostatic hypotension (IOH) (3.08-fold), reflecting this autonomic impairment. Altered catecholamine dynamics—specifically, increased norepinephrine from reduced clearance but blunted epinephrine secretion—impair stress responses, increasing the risk of orthostatic hypotension, arrhythmias, and cardiovascular events (29).

Sympathetic overactivation with reduced parasympathetic tone promotes cardiac remodeling, arrhythmia risk, and sudden cardiac death. This autonomic imbalance exacerbates inflammation, fibrosis, and electrical instability while impairing cardiac homeostasis (30).

Concurrently, inflammation resulting from cortisol-DHEA imbalance accelerates endothelial dysfunction and atherosclerosis, depleting physiological reserve. The resulting vulnerability to stressors like infection or surgery reinforces the bidirectional frailty-CVD link, highlighting neuroendocrine pathways as promising therapeutic targets (28).

##### 2.1.1.5 Interplay of shared mechanisms: a vicious cycle

The mechanisms described in the above sections interact synergistically to create a vicious cycle of physiological decline. This interplay is fundamental to understanding the synergy between frailty and CVD. For instance, Chronic inflammation directly damages the endothelium, promotes mitochondrial ROS production, and disrupts neurohormonal axes. Conversely, mitochondrial dysfunction amplifies oxidative stress, which further fuels inflammation, worsens endothelial function, and activates stress pathways like the RAAS. Similarly, endothelial dysfunction causes hypoperfusion, exacerbating mitochondrial failure and sarcopenia, while also promoting a pro-inflammatory and pro-thrombotic state. Neurohormonal activation, in turn, exacerbates inflammation, oxidative stress, and endothelial dysfunction, while simultaneously impairing mitochondrial function. Within this interconnected network, a perturbation in one system rapidly affects the others, triggering a cascade of multisystem dysfunction. This explains the accelerated functional decline seen in patients with coexisting frailty and CVD and underscores why therapeutic strategies targeting single pathways may have limited success compared to multimodal interventions that address this integrated pathophysiology.

#### 2.1.2 Specific interaction mechanisms

The shared biological mechanisms create a vulnerable physiological state; however, the clinical synergy between frailty and CVD is driven by specific, bidirectional pathways. These direct interactions explain how the phenotypic features of one condition can directly precipitate or exacerbate the other, as detailed in the following sections.

##### 2.1.2.1 Muscle‒heart axis

Sarcopenia, the depletion of skeletal muscle mass and strength that is a hallmark of frailty, directly impacts cardiovascular health. This reduction in musculature leads to decreased physical activity, systemic deconditioning, and increased cardiac workload. Sarcopenia is associated with impaired cardiac function and reduced exercise capacity, including reduced peak oxygen uptake and stroke volume during exercise. While mechanisms such as impaired venous return and diminished peripheral oxygen utilization require further elucidation, these changes underscore sarcopenia's significant contribution to cardiovascular decline. The resulting cycle of deconditioning worsens clinical outcomes, emphasizing the necessity of muscle-preserving interventions in aging adults (31, 32).

##### 2.1.2.2 Metabolic-frailty-cardiovascular axis

Metabolic dysregulation serves as a critical link between frailty and CVD through interconnected pathways of insulin resistance, chronic inflammation, and dysfunctional lipid metabolism (33). Frail individuals frequently exhibit impaired glucose homeostasis and altered adipokine secretion, which fosters a proatherogenic milieu that accelerates vascular dysfunction (34). A key mediator of this relationship is visceral adiposity, particularly epicardial adipose tissue (EAT), which secretes proinflammatory cytokines that promote myocardial fibrosis, endothelial dysfunction, and plaque instability (35). Clinically, increased EAT volume independently predicts major adverse cardiovascular events (MACEs), CAD, AF, and HF, with particularly strong associations in frail older adults (34, 36).

The sarcopenic obesity phenotype exemplifies this metabolic-frailty-CVD axis, combining ectopic fat accumulation with muscle depletion to create a proinflammatory and proatherogenic state (37). This phenotype drives simultaneous muscle catabolism and cardiac remodeling through collagen deposition, ventricular stiffening, and diastolic dysfunction, while also accelerating the progression of coronary atherosclerosis (38, 39).

Mitochondrial dysfunction and oxidative stress are key mediators of these pathological changes, impairing energy metabolism in both cardiac and skeletal muscle. This ultimately reduces physiological reserve and reinforces the bidirectional frailty-CVD relationship (40).

##### 2.1.2.3 Nutritional deficiency and anabolic resistance

Nutritional deficiencies and anabolic resistance represent key pathological mechanisms in frailty, affecting 15%–50% of community-dwelling older adults through multifactorial causes including age-related anorexia and impaired nutrient absorption (41). This nutritional deprivation is compounded by anabolic resistance, a state in which skeletal muscle shows a blunted protein synthesis response to nutritional and hormonal stimuli (42). Chronic inflammation contributes to this process by suppressing the mechanistic target of rapamycin (mTOR) signaling pathway and promoting muscle atrophy (43), while mitochondrial dysfunction limits energy availability for tissue repair (40). These effects are exacerbated by age-related declines in anabolic hormones insulin-like growth factor-1 (IGF-1), testosterone and growth hormone, which impair muscle and cardiac maintenance (44, 45).

Micronutrient deficiencies significantly impact both frailty and CVD progression. Vitamin D deficiency impairs calcium metabolism and cardiovascular health through endothelial dysfunction and RAAS activation (46), while inadequate vitamin B12 and folate elevate homocysteine, thereby promoting vascular damage and atherosclerosis (47).

Antioxidant deficiencies (e.g., vitamins C and E) increase oxidative endothelial damage, and insufficient magnesium and potassium contributes to hypertension and arrhythmias (48–50). These micronutrient deficiencies significantly impair immune function, exacerbating frailty and elevating cardiovascular risk. These nutritional deficits collectively accelerate physical and cardiovascular decline by disrupting essential metabolic processes, promoting endothelial dysfunction, and amplifying systemic inflammation. The resulting multisystem deterioration highlights nutrition as a critical modifiable factor in preserving healthspan and mitigating frailty-related complications in aging populations.

##### 2.1.2.4 Pharmacokinetic changes and their impact on cardiovascular therapy

Frailty alters drug pharmacokinetics through multiple age-related changes. Delayed gastric emptying and reduced gastric motility can lead to delayed absorption and reduced bioavailability of some orally administered drugs (51). Changes in the gut microbiome further alter drug bioavailability and metabolism (52). Sarcopenia and increased adiposity in frail individuals alter the volume of distribution, increasing it for lipophilic drugs and decreasing it for hydrophilic drugs (53). Reduced plasma albumin levels in frail individuals decrease protein binding for acidic drugs, whereas chronic inflammation reduces the expression of drug-metabolizing enzymes and transporters, increasing protein binding for steroids and neutral or basic drugs due to elevated alpha-1 acid glycoproteins (54). Aging and frailty reduce hepatic volume and blood flow, impairing both phase I and phase II hepatic clearance, and a concurrent decline in the glomerular filtration rate reduces renal clearance, thereby compounding the risk of drug accumulation and toxicity (54).

These changes particularly impact cardiovascular drugs: impaired renal function potentiates Angiotensin-converting enzyme (ACE) inhibitor and β-blocker toxicity, while impaired metabolism can decreases statin efficacy. Consequently, frail patients show higher adverse drug reaction rates (55).

##### 2.1.2.5 Vascular repair deficiency and stem cell aging

The decline in vascular repair capacity with aging and frailty is characterized by significant reductions in the number and function of EPCs, which are essential for maintaining endothelial integrity and promoting angiogenesis (17). This impairment stems from several interrelated mechanisms, including stem cell exhaustion due to accumulated DNA damage and epigenetic changes (56), telomere attrition leading to proliferative arrest (57), and diminished angiogenic signaling through pathways such as vascular endothelial growth factor (VEGF) and stromal cell-derived factor-1/C-X-C Chemokine Receptor Type 4 (SDF-1/CXCR4) (58, 59). These deficits contribute to arteriosclerosis, capillary rarefaction, and tissue hypoxia, creating a vicious cycle that exacerbates both cardiovascular dysfunction and frailty. Hypoxia further compromises EPC function by destabilizing hypoxia-inducible factor 1-alpha (HIF-1α), impairing adaptive responses to ischemia (60). Clinically, this manifests as increased arterial stiffness, reduced exercise tolerance, and heightened vulnerability to ischemic events.

**Figure 1:**
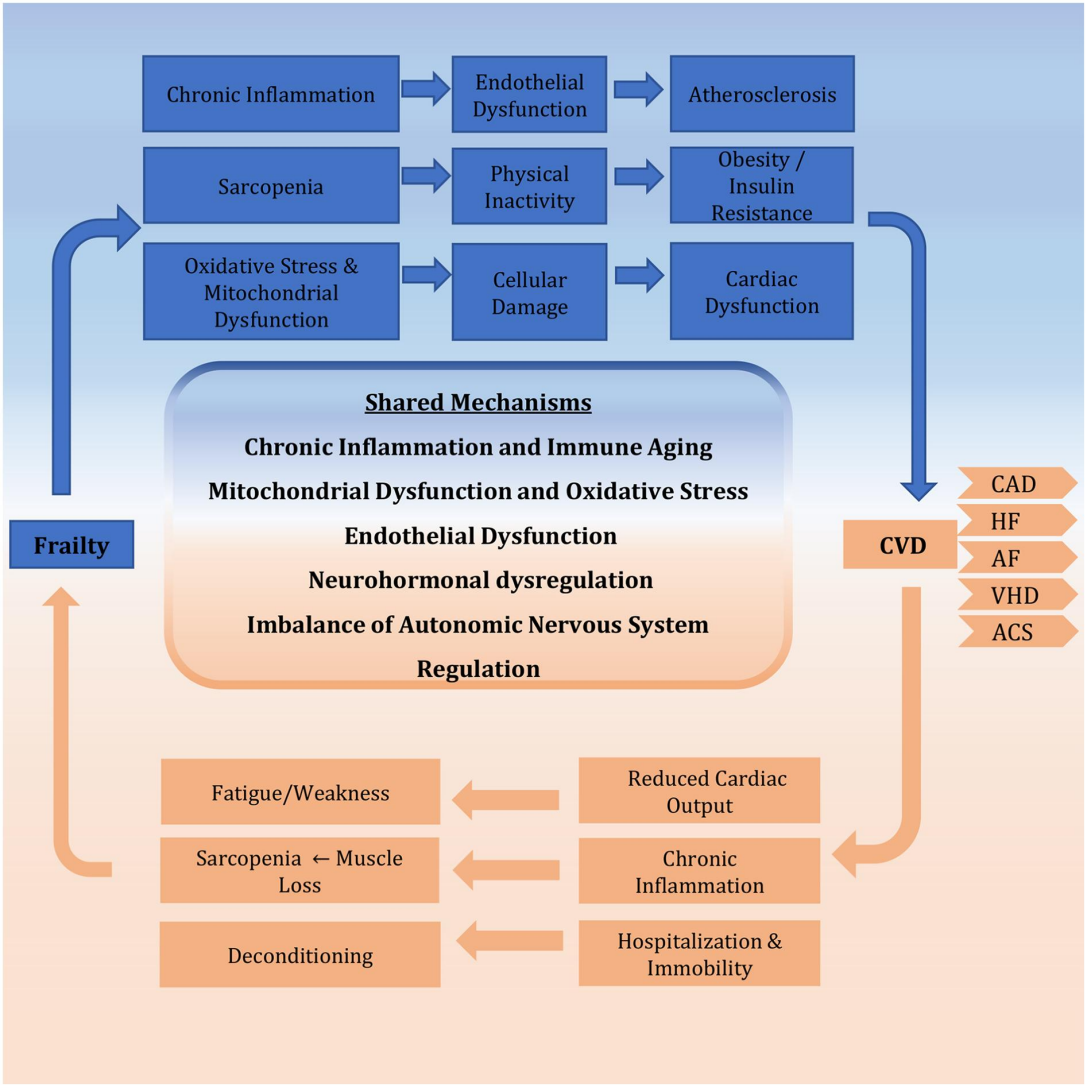
Bidirectional relationship between frailty and CVD. The schematic illustrates the vicious cycle whereby frailty and CVD mutually reinforce each other. This synergy is driven by core shared mechanisms, including chronic inflammation, mitochondrial dysfunction, neurohormonal dysregulation, and endothelial dysfunction, which collectively accelerate multisystem decline. CVD, cardiovascular disease; CAD, coronary artery disease; HF, heart failure; AF, atrial fibrillation; VHD, valvular heart disease; ACS, acute coronary syndrome.

This section has examined the shared biological pathways linking frailty and CVD, demonstrating how these conditions mutually reinforce one another through interconnected mechanisms. Chronic low-grade inflammation, marked by elevated IL-6 and TNF-α, drives both muscle wasting in frailty and endothelial dysfunction in CVD. Mitochondrial deterioration similarly impacts cardiac function and skeletal muscle through oxidative stress and energy deficits. Neurohormonal imbalances contribute to cardiovascular remodeling while reducing physiological resilience. Vascular dysfunction emerges as a critical bidirectional link, with impaired endothelial repair and progenitor cell activity exacerbating tissue hypoperfusion and atherosclerosis progression. Autonomic nervous system dysregulation further reduces cardiovascular adaptability. These interconnected pathways create a framework where cellular damage, metabolic dysregulation, and impaired repair capacity mutually reinforce frailty and CVD (Figure 1). This analysis reveals these conditions represent converging manifestations of fundamental aging processes rather than simple comorbidity, explaining their frequent coexistence in elderly populations. To summarize the core pathophysiological pathways and their clinical implications, Table 1 provides a concise overview of the key mechanisms and links them to their clinical manifestations and potential interventional targets.

**Table 1:** Core pathophysiological mechanisms linking frailty and cardiovascular disease: clinical consequences and potential interventions.

Mechanisms	Key mediators/pathways	Clinical consequences in CVD	Clinical consequences in frailty	Potential targeted interventions
Chronic inflammation (inflammaging)	IL-6, TNF-α, NLRP3 inflammasome (IL-1β, IL-18)	• Atherosclerotic plaque progression & instability • Endothelial dysfunction • Prothrombotic state	• Sarcopenia (muscle wasting) • Anabolic resistance • Fatigue & functional decline	• Lifestyle (structured exercise, Mediterranean diet) • Canakinumab (IL-1β inhibition) • Nutritional support (omega-3, protein)
Mitochondrial dysfunction & oxidative stress	↓ ATP production, ↑ ROS, impaired mitophagy (Drp1, PGC-1α)	• Impaired cardiac contractility (Heart Failure) • Oxidative endothelial damage • Vascular inflammation	• Skeletal muscle bioenergetic failure • Accelerated proteolysis & weakness • Exercise intolerance	• Exercise training (improves biogenesis) • Mitochondrial antioxidants (CoQ10, MitoQ) • Metabolic modulators (e.g., NAD + precursors)
Neurohormonal dysregulation	•RAAS overactivation (Angiotensin II) • HPA axis imbalance (↑Cortisol/↓DHEA) • ANS dysfunction (↑Sympathetic tone)	• Vascular fibrosis & stiffness • Cardiac remodeling • Hypertension, arrhythmias	• Muscle catabolism • Metabolic dysfunction • Reduced physiological reserve	• RAAS inhibitors (ACEi, ARBs, MRAs) • Beta-blockers • Physical activity & stress reduction
Endothelial dysfunction	↓ NO bioavailability, ↑ ADMA, ↑ arterial stiffness, ↓ VEGF signaling	• Atherosclerosis progression • Plaque vulnerability • Impaired vasodilation	• Tissue hypoperfusion • Exacerbates sarcopenia & mitochondrial dysfunction	• ACE inhibitors/ARBs • Exercise (increases NO) • Senolytics (improve EPC function) • Angiogenic gene therapy (VEGF)

This table summarizes the key shared biological pathways, their clinical manifestations in both conditions, and emerging therapeutic strategies.

CVD, cardiovascular disease; IL-6, Interleukin-6; TNF-α, Tumor Necrosis Factor-alpha; ATP, Adenosine Triphosphate; ROS, Reactive Oxygen Species; Drp1, Dynamin-related protein 1; PGC-1α, Peroxisome proliferator-activated receptor gamma coactivator 1-alpha; RAAS, Renin-Angiotensin-Aldosterone System; HPA, Hypothalamic-Pituitary-Adrenal; ANS, Autonomic Nervous System; DHEA, Dehydroepiandrosterone; NO, Nitric Oxide; ADMA, Asymmetric Dimethylarginine; VEGF, Vascular Endothelial Growth Factor; EPC, Endothelial Progenitor Cell; VO₂, Oxygen Uptake; IGF-1, Insulin-like Growth Factor 1; GM-CSF, Granulocyte-Macrophage Colony-Stimulating Factor.

### 2.2 Assessment of frailty

Frailty assessment has evolved significantly since the introduction of the Fried Frailty Phenotype in 2001, with nearly 70 tools now available measuring physical, psychological, and social dimensions. Despite this proliferation, no universal standard exists for frailty assessment in CVD management, where it critically impacts outcomes and treatment decisions (61, 62).

Current tools fall into several categories (Table 2), one of them being clinical scales which typically use an ordinal system ranging from fit to severely frail to quickly assess patients in a clinical setting. These include tools such as the Fried Frailty Phenotype, which assesses physical parameters such as weight loss, exhaustion, activity, grip strength, and gait speed are assessed (61) and the Clinical Frailty Scale (CFS), a 9-point visual scale integrating function and cognition. Their rapid administration makes them valuable for clinical settings. The Fried phenotype, while predictive, is less practical in clinical practice as it does not incorporate cognitive/psychosocial factors (63). Meanwhile, the CFS is highly practical for busy clinical environments. Widely used globally, it is most prevalent in Canada and United Kingdom but has also been adopted in Asia, South America, and other parts of Europe (64). The CFS is frequently employed to predict health outcomes, such as mortality, comorbidity, functional decline, mobility, and cognitive decline, further underscoring its utility as a promising frailty screening tool.

**Table 2:** Frailty assessment tools – general and cardiac-specific comparisons.

Tool	Description	Strengths	Limitations	Clinical application
Fried Phenotype (General)	Assesses 5 criteria: unintentional weight loss, self-reported exhaustion, low physical activity, weak grip strength, slow gait speed.	Predicts adverse outcomes; widely validated in epidemiological research.	Excludes cognitive/psychosocial dimensions; requires objective performance measures.	Research studies; screening of community-dwelling older adults without known cardiovascular disease.
Clinical Frailty Scale (CFS) (General)	9-point pictorial and descriptive scale (1 = very fit to 9 = terminally ill) integrating comorbidity, function, and cognition.	Very quick (<2 min); combines functional and cognitive assessments.	Subjective; may lack precision in acutely ill hospitalized patients.	Rapid clinical triage in emergency departments and hospital admission units.
Frailty Index (FI) (General)	Quantifies frailty as a ratio of health deficits present to total deficits considered (score range 0–1).	Highly comprehensive; sensitive to gradual frailty progression.	Time-consuming (requires 30 + clinical variables); needs extensive data collection.	Longitudinal aging studies; comprehensive geriatric assessments in outpatient clinics.
Electronic Frailty Index (e-FI) (General: Automated)	36-item tool based on Rockwood's deficit accumulation model, calculated from routine primary care data.	Highly scalable for population health; predicts hospitalization and mortality.	Limited to specific healthcare systems (e.g., UK primary care) due to dependency on specific Electronic Health Record coding.	Large-scale screening and risk stratification in primary care populations (e.g., UK).
Electronic Screening Instrument for Frailty (e-SIF) (General: Automated)	Automated frailty screening tool generating immediate results from available patient data.	Provides immediate results; predicts mortality and hospitalization.	Requires full Electronic Health Record integration and interoperability; has limited external validation.	Automated screening of hospitalized patients within health systems with advanced digital infrastructure.
Comprehensive Assessment of Frailty (CAF) (Cardiac-Specific)	Combines physical tasks (chair stands, stair climbing), self-reported weakness, and serum creatinine level.	Objective performance measures; validated in cardiac surgery populations.	Requires performance testing space and time; not validated in non-surgical cardiovascular disease populations.	Preoperative risk stratification for patients undergoing cardiac surgery (e.g., Coronary Artery Bypass Grafting).
Modified Fried Criteria (Cardiac-Specific)	Adapts the Fried Phenotype with gait speed, handgrip strength, Activities of Daily Living dependence, and serum albumin level.	Adapts a well-known model for cardiovascular disease populations.	Limited data on predictive validity for specific cardiovascular outcomes.	Frailty assessment in older adults with established cardiovascular disease in outpatient cardiology settings.
Essential Frailty Toolset (EFT) (Cardiac-Specific)	Assesses standing balance (timed chair rise), cognition (Mini-Cog test), hemoglobin, and serum albumin.	Strongly predicts mortality and morbidity in cardiac surgery; very simple (4 items).	Not validated in non-surgical cardiovascular disease populations (e.g., chronic heart failure).	Preoperative risk stratification specifically for patients being evaluated for Transcatheter Aortic Valve Replacement or cardiac surgery.
Tilburg Frailty Indicator (TFI) (Cardiac-Specific)	15-item self-reported questionnaire covering physical, psychological, and social domains of frailty.	Holistic; captures important psychosocial components of frailty.	Subjective; may overestimate frailty in patients with depression.	Comprehensive frailty evaluation in outpatient chronic disease management, including heart failure clinics.

Comparison of commonly used general and cardiac-specific frailty assessment tools. General tools are broadly applicable, while Cardiac-specific tools are designed or validated for use in populations with cardiovascular disease, often incorporating disease-relevant metrics. The Clinical Application column provides guidance on the most appropriate settings for each tool's use.

CFS, Clinical Frailty Scale; FI, Frailty Index; e-FI, Electronic Frailty Index; e-SIF, Electronic Screening Instrument for Frailty; CAF, Comprehensive Assessment of Frailty; EFT, Essential Frailty Toolset; TFI, Tilburg Frailty Indicator; CVD, Cardiovascular Disease; ADL, Activities of Daily Living; HER, Electronic Health Record; TAVR, transcatheter aortic valve replacement; CABG, Coronary Artery Bypass Grafting.

The next category is composite indices, an example is the Frailty Index (FI) which quantifies health deficits across physical, psychological, and social domains, providing continuous scoring (0–1). This scoring system offers a nuanced and precise measure of frailty severity, capturing the gradual progression of health decline rather than categorizing individuals into binary states of “frail” or “non-frail”. Although comprehensive, its data requirements limit its routine use. Frailty indices are especially useful in research and longitudinal studies, as they offer a more nuanced and sensitive measurement of frailty progression over time (65). Despite these limitations, FI has proven useful for predicting adverse outcomes, such as increased mortality and reduced life expectancy, and has been proposed as a tool for planning health services by Sternberg et al. (66).

The third category is the electronic tools where automated indices like Electronic Frailty Index (e-FI) a 36-item tool based on Rockwood's deficit accumulation model and Electronic Screening Instrument for Frailty (e-SIF) enable efficient screening but face adoption barriers outside of certain countries due to coding differences (67).

Finally, the use of biomarkers such as inflammatory markers, hormones and oxidative stress indicators show promise but they lack standardization. Hormonal changes, such as decreases in testosterone and elevated cortisol levels, as well as increased oxidative stress, further highlight the complex interplay of the biological processes driving frailty (68). When combined with clinical tools these biomarkers may improve early detection and help in understanding their impacts on patient prognosis.

The need for a cardiac-specific frailty assessment tool arises from the high prevalence of frailty among older adults with CVD and its significant impact on clinical outcomes (69). Frailty in cardiac patients is associated with increased mortality, complications after surgeries such as TAVR or cardiac surgery, and longer hospital stays, necessitating a tailored approach for assessment (70, 71). Cardiac specific tools which assess patients with tailored scaled which account for CVD-specific measures such as NT-proBNP levels, the 6-minute walk distance, or HF symptoms, these tools enhance predictive accuracy and support shared decision-making, ensuring that care aligns with patient goals (Table 2).

Several validated tools exist such as the Comprehensive Assessment of Frailty (CAF) which evaluates frailty through physical tasks, self-reported weakness, and serum creatinine levels. Scores range from 1 to 35, with 1–10 indicating no frailty, 11–25 indicating moderate frailty, and 26–36 indicating severe frailty (72). Green et al. (73) applied the modified Fried frailty criteria, which incorporate gait speed, handgrip strength, ADL, and serum albumin levels. Frailty is defined as a score >5 on a 0–12 scale, with higher scores indicating greater frailty. Afilalo et al. (74) utilized four scales: the 5-item and 7-item modified Fried criteria, the 4-item MacArthur Study of Successful Aging (MSSA), and the Five-Meter gait speed test. Frailty is identified if any scale deems the patient frail. Schoenenberger et al. (75) employed a multidimensional geriatric assessment that combines cognitive impairment (Mini-Mental State Examination, MMSE), malnutrition (Mini Nutritional Assessment, MNA), mobility (Timed Up and Go test, TUG), and ADL limitations. Frailty is defined as a score of ≥3 points, with additional points for specific deficits. Jung et al. (76) used the Modified Fried Frailty Criteria and a 35-item Frailty Index, which includes comorbidities, physical and emotional measures, and functional limitations. Frailty is determined by a score based on the proportion of deficits present. The Short Physical Performance Battery (SPPB) was also used, with frailty defined as a composite score ≥9. Uchmanowicz et al. (77) developed the Tilburg Frailty Indicator (TFI), which assesses physical, psychological, and social domains through 15 self-reported questions. A score >5 indicates frailty. Dunlay et al. (78) created a 31-item deficit index, categorizing patients into tertiles (lowest = not frail, middle = intermediate frail, highest = frail). Finally, Afilalo et al. (79) introduced the Essential Frailty Toolset, which evaluates standing time, cognition, hemoglobin, and serum albumin, with a composite score of 0–5 indicating frailty. These tools highlight the multidimensional nature of frailty and its critical role in predicting outcomes in CVD.

Ultimately, a cardiac-specific frailty tool optimizes outcomes, reduces complications, and improves the quality of life for this vulnerable population by embedding frailty assessment into routine cardiovascular care. Frailty assessment is increasingly recognized as a critical component of preoperative evaluation for cardiac surgery. Identifying frail patients helps stratify surgical risk, guide decision-making, and optimize perioperative care.

Integrating these assessments into cardiovascular care improves risk prediction, particularly for surgical candidates who may benefit from prehabilitation. Future development should focus on standardized, disease-specific tools to optimize management of frail cardiac patients.

### 2.3 Epidemiological evidence

**Figure 2:**
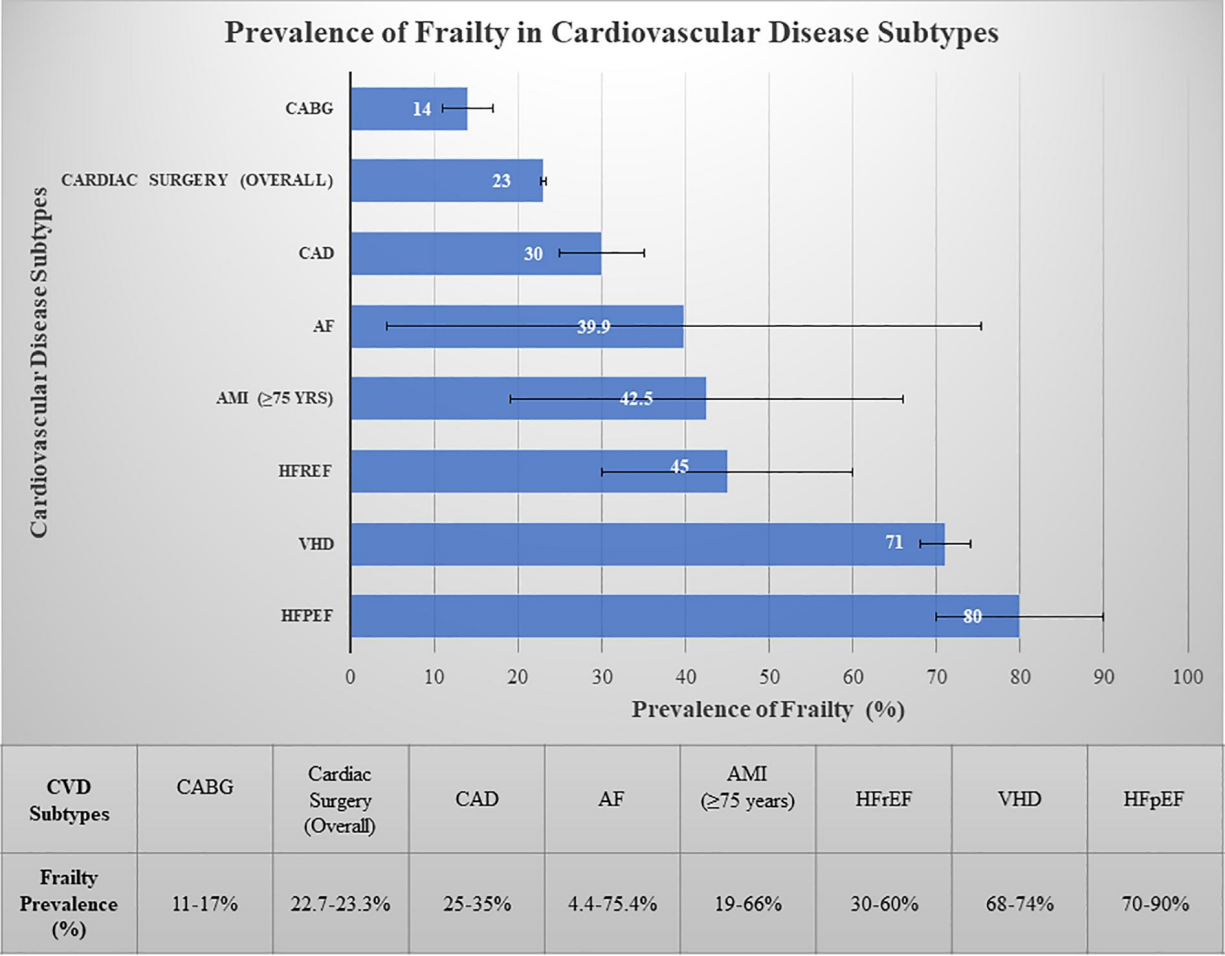
Prevalence of frailty in cardiovascular disease subtypes. Bars represent midpoint estimates of frailty prevalence. Horizontal error bars indicate the range of reported values from systematic reviews and meta-analyses. The substantial variability for conditions such as atrial fibrillation reflects heterogeneity in assessment tools (e.g., lower prevalence with the Clinical Frailty Scale vs. higher prevalence with the Frailty Index), patient age (e.g., significantly higher prevalence in patients ≥80 years), and clinical settings. HFpEF, heart failure with preserved ejection fraction; HFrEF, heart failure with reduced ejection fraction; AMI, acute myocardial infarction; AF, atrial fibrillation; CAD, coronary artery disease; VHD, valvular heart disease; CABG, coronary artery bypass grafting.

**Figure 3:**
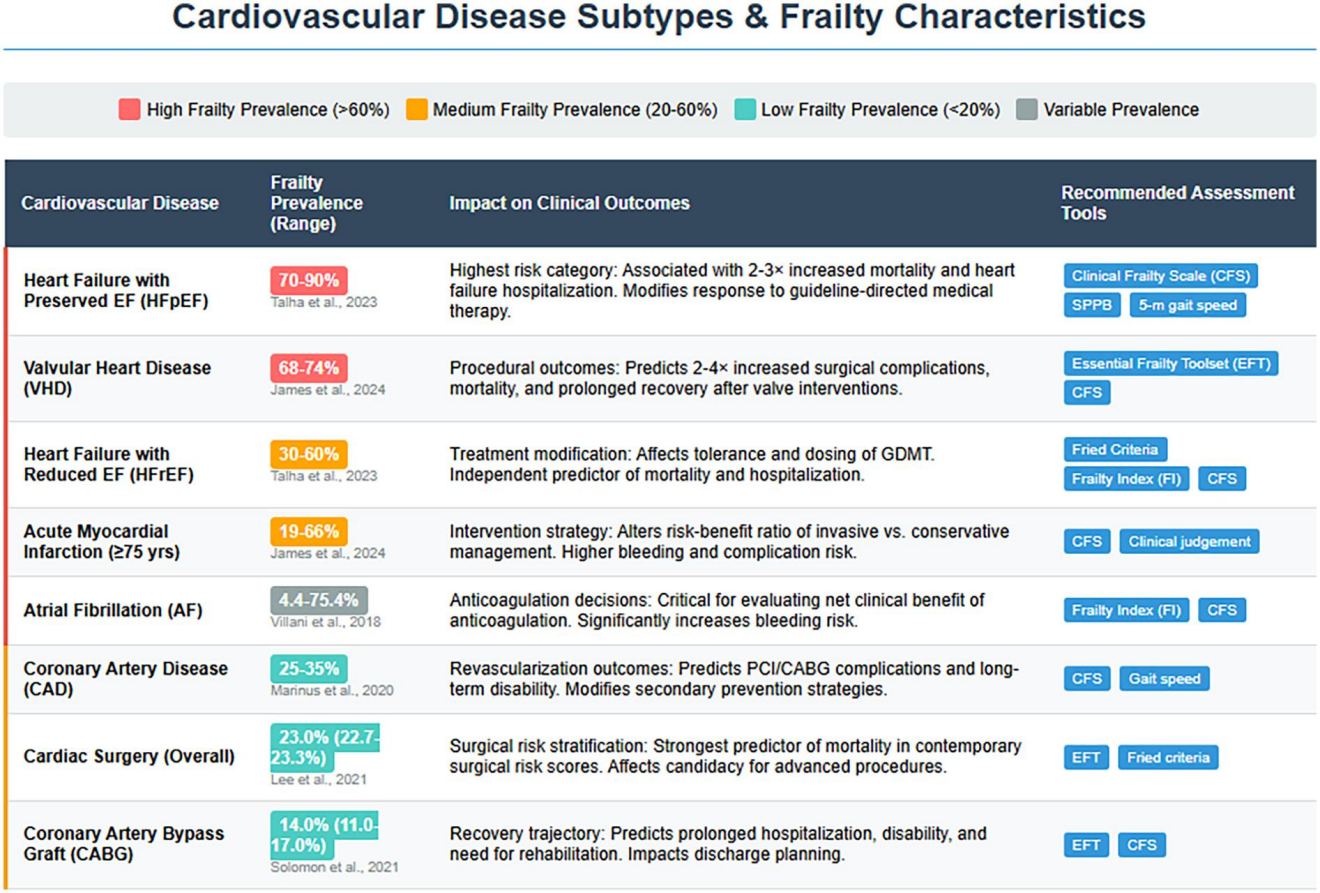
Frailty characteristics and clinical implications across cardiovascular disease subtypes: frailty prevalence ranges are categorized by color intensity (dark red: high burden >60%; orange: medium burden 20%–60%; teal: low burden <20%; gray: variable burden). The substantial prevalence in conditions such as HFpEF and VHD underscores frailty as a central determinant of clinical outcomes rather than merely a comorbid condition. Condition-specific assessment tools are recommended to guide management decisions across the cardiovascular spectrum. HFpEF, heart failure with preserved ejection fraction; HFrEF, heart failure with reduced ejection fraction; AMI, acute myocardial infarction; AF, atrial fibrillation; CAD, coronary artery disease; VHD, valvular heart disease; CABG, coronary artery bypass graft; CFS, Clinical Frailty Scale; SPPB, Short Physical Performance Battery; EFT, Essential Frailty Toolset; FI, Frailty Index; GDMT, guideline-directed medical therapy; PCI, percutaneous coronary intervention.

Frailty prevalence varies significantly among older adults with CVD, ranging from 14% in patients undergoing coronary artery bypass grafting (CABG) to 80% in those with HF with preserved ejection fraction (HFpEF) patients, 74% in aortic valve disease and 4.4%–75.4% in AF depending on assessment tools (Figure 2) (80, 81). Notably, frailty is more common in women, with a prevalence approximately 1.6 times higher than in men. As illustrated in our association diagram (Figure 3), frailty also worsens CVD outcomes, increasing mortality risk 2.5–3.5-fold post- percutaneous coronary intervention (PCI) (80) and is associated with hazard ratios of 1.77 for MACE, 1.95 for AMI, and 1.71 for stroke (82).

Frailty increases the risk of peripheral vascular disease (PVD) by 1.80-fold and CAD by 1.35-fold and is also associated with a 1.6-fold and 2.6-fold increased risk of fatal CVD (69).

The clinical implications of these prevalence patterns (Figures 2, 3) are substantial, with frail acute coronary syndrome (ACS) patients showing 1.54–5.39-fold higher mortality. Furthermore, older frail individuals were significantly less likely to receive guideline-recommended ACS treatments, such as PCI. The rates of PCI in frail patients ranged from 6.7% to 43.7%, whereas they ranged from 30.4% to 69.5% in non-frail patients (83). Frail ACS patients also experience higher in-hospital mortality, major bleeding, and stroke, along with longer hospital stays and increased rates of disability and readmission.

Patients who undergo CABG and who are frail tend to experience extended hospital stays, which increases their likelihood of developing disabilities, requiring subsequent hospitalizations, and increasing mortality rates (84). Frailty identified by the EFT was associated with a 3-fold increase in all-cause mortality post-CABG (85).

In a comprehensive review by Talha, K. M., et al., frailty was shown to impact at least half of all HF patients, particularly in patients with HFpEF, affecting up to 90% of this population (Figure 2), whereas it was 30%–60% in those with HF with reduced ejection fraction (HFrEF). Frailty was seen to be approximately 26% more common in women than in men. Patients with HF have an ∼20% greater prevalence of sarcopenia than those without HF (86).

Valvular heart disease (VHD) prevalence increases with age, affecting 0.7% of those under 45 years to upto 13.3% in those over 75 years (87). VHD causes significant hemodynamic changes increasing the risk of MACEs (79), with severe aortic stenosis predicting mortality and mitral regurgitation worsening outcomes in geriatric patients (88). Frailty affects up to 71% of older VHD patients, exacerbating medication intolerance, procedural complications, mortality, and functional/cognitive decline (Figure 3) (79). Interventions such as percutaneous mitral valve repair and TAVR can reduce the prevalence of frailty and improve quality of life, depression, and functional outcomes (89, 90).

### 2.4 Integrated management and emerging therapeutics

The clinical management of frailty in older adults with CVD requires a multimodal approach that addresses physical, nutritional, and cognitive domains. This section outlines the evolution from traditional challenges to contemporary and emerging strategies for managing this vulnerable population.

#### 2.4.1 Challenges in risk assessment and treatment disparities

One of the most significant challenges in managing CVD in older adults is the risk assessment dilemma. Traditional risk assessment tools, such as EuroSCORE II, often fail to account for the complexities of frailty, leading to inaccurate risk stratification. These tools focus primarily on chronological age and comorbidities but overlook the physiological decline and vulnerability associated with frailty (74, 91). This creates a paradox in treatment decision-making. Frail patients are less likely to receive guideline-recommended interventions, such as PCI or CABG, with utilization rates ranging from 6.7% to 43.7% compared to 30.4% to 69.5% in non-frail patients, highlighting a significant treatment disparity (83). Consequently, this underutilization may contribute to poorer outcomes. Furthermore, shared decision-making is complicated by the fact that older frail patients with non-ST elevation ACS have higher rates of refusal for invasive procedures like coronary angiography. This choice, while reflecting patient preference, is associated with a near doubling of the risk for long-term mortality (Hazard Ratio: 1.97), underscoring the complexity of risk-benefit discussions in this population (92). For those who undergo procedures, the psychological adjustment to cardiac implantable electronic devices (CIEDs) is significantly influenced by age. Contrary to assumptions, while quality of life more often improves in older patients (>75 years), younger patients (≤75 years) experience a greater psychological burden, reporting more difficulties in their professional and private lives and feeling more limited by the device (93). This finding underscores the need for age-tailored psychosocial support.

#### 2.4.2 Implementation of foundational interventions: from rehabilitation to integrated care

This section details the practical implementation of foundational interventions, focusing on structured exercise and nutritional programs within collaborative care models to translate evidence into improved outcomes for frail patients with CVD.

##### 2.4.2.1 Exercise as a cornerstone therapy with proven efficacy

Structured physical activity is far more than a simple recommendation for functional improvement; it is a cornerstone, evidence-based therapeutic strategy for managing both frailty and CVD. Its unique efficacy stems from its role as a powerful pleiotropic intervention, capable of simultaneously targeting the fundamental biological pathways of aging that drive these conditions. As powerfully summarized by Angulo et al. (94), the protective effects of exercise are mediated through a concert of interconnected molecular and cellular mechanisms that counteract the core drivers of aging. Exercise reduces age-related oxidative damage and chronic inflammation, while increasing autophagy, improving mitochondrial function, modulating the myokine profile, and restoring the IGF-1 signaling pathway and insulin sensitivity. These mechanisms form the scientific foundation for exercise's clinical benefits.

The aging process is characterized by a rise in reactive oxygen species (ROS) and a state of low-grade chronic inflammation, or “inflammaging” (95). Exercise counteracts these dual threats. It activates the transcription factor Nrf2 (96), boosting the expression of antioxidant enzymes like catalase and superoxide dismutase to mitigate oxidative damage (97). Concurrently, regular exercise reduces the expression of pro-inflammatory pathways [e.g., toll-like receptors (TLR4) on monocytes] and lowers levels of cytokines like IL-6 and TNF-α (98), while increasing anti-inflammatory mediators such as IL-10 and adiponectin. This evidence confirms that age-related oxidative stress and inflammation are modifiable with structured physical activity (94, 99).

Enhancement of autophagy and mitochondrial function is a key mechanism by which exercise counteracts sarcopenia by revitalizing cellular quality control. Autophagy, the process of clearing damaged cellular components, is impaired in aged muscle. Exercise modulates key autophagy markers like LC3II, promoting the removal of dysfunctional proteins and organelles (100). This is intrinsically linked to improved mitochondrial health. Through the upregulation of PGC-1α, the master regulator of mitochondrial biogenesis (101), exercise enhances mitochondrial function and promotes mitophagy (the selective autophagy of damaged mitochondria), thereby combating the mitochondrial dysfunction that underlies muscle fatigue and wasting (101). For example, long-term exercise increases the LC3II/I ratio, a marker of autophagy, and prevents sarcopenia in aged models, while lifelong exercise helps preserve autophagic and mitophagic capacity (94, 102).

Aging impairs the insulin/IGF-1 signaling pathway, leading to insulin resistance and diminished muscle protein synthesis via the PI3K/AKT/mTOR pathway. Lower IGF-1 levels are independently associated with frailty (103). Exercise directly addresses this deficit. It improves insulin sensitivity by increasing glucose uptake into muscles (104). Furthermore, resistance training, in particular, activates the IGF-1/mTOR pathway, stimulating muscle protein synthesis (105). This restoration of metabolic and anabolic signaling is crucial for maintaining muscle mass and function (94).

Skeletal muscle acts as an endocrine organ, releasing myokines in response to contraction. Exercise beneficially alters this myokine profile. It increases the release of irisin (associated with metabolic health) and decorin (which inhibits the muscle-wasting protein myostatin) (106), while restoring levels of apelin, a myokine involved in muscle regeneration (107). This improved myokine signaling provides a systemic explanation for how localized muscle activity can produce body-wide benefits, directly contributing to the reduction of age-related muscle loss and dysfunction (94, 108).

The robust mechanistic evidence for exercise is strongly supported by landmark clinical trials. The LIFE study demonstrated that a structured physical activity program significantly reduced the risk of major mobility disability in sedentary older adults (109). Similarly, the VIVIFRAIL program showed that multicomponent exercise interventions—combining strength, balance, and endurance training—led to significant improvements in gait speed, physical performance, and frailty status (110). These trials confirm that the profound molecular benefits of exercise, as detailed by Angulo et al., translate directly into meaningful clinical outcomes, solidifying its role as an indispensable strategy for healthy aging.

##### 2.4.2.2 The synergistic role of nutritional support

Nutritional interventions play a critical role in combating sarcopenia and anabolic resistance. Specifically, protein and vitamin D supplementation have been shown to improve muscle mass, strength, and physical performance (111). The SPRINT-T trial demonstrated that a multimodal approach is particularly effective, showing that combining exercise with nutritional support led to significant gains in muscle mass and a reduction in overall frailty prevalence (112). This synergy underscores the power of an integrated management strategy.

##### 2.4.2.3 Delivery through structured rehabilitation programs

The efficacy of exercise and nutrition is best realized through structured rehabilitation programs. Cardiac rehabilitation has evolved to incorporate explicit frailty management. In recognition of this, the 2021 European Society of Cardiology (ESC) guidelines now give a Class IIA recommendation for supervised, exercise-based cardiac rehabilitation specifically for patients with advanced disease, frailty, and multiple comorbidities (113).

Cardiac telerehabilitation (CTR) has emerged as a vital delivery model, particularly for increasing accessibility. CTR combines the benefits of traditional rehabilitation with remote supervision, improving physical function (e.g., 6-minute walk distance) and psychological well-being, with demonstrated safety and high completion rates in clinical trials (114). While challenges like technological literacy persist, CTR represents a critical innovation for delivering these foundational interventions to vulnerable populations at scale.

##### 2.4.2.4 The multidisciplinary geriatric cardiology model

To comprehensively address the complex needs of frail patients, the field has embraced multidisciplinary team-based care. This geriatric cardiology model, brings together cardiologists, geriatricians, physiotherapists, and dietitians in a collaborative framework. Within this framework, foundational interventions like personalized exercise and nutritional plans are embedded within a broader strategy that includes comprehensive geriatric assessment, pre-procedural optimization, and post-discharge support, ultimately leading to improved outcomes (115).

#### 2.4.3 Emerging therapeutic strategies for frailty and CVD (477 words)

Innovative therapeutic strategies are being explored to improve outcomes in frail older adults with CVD. These strategies aim to restore vascular repair capacity, which is often impaired in individuals with frailty and CVD, and include the following:

##### 2.4.3.1 Senolytic therapy

Senolytic agents such as dasatinib and quercetin have shown promising results in improving EPC function (116). An ongoing phase II trial (NCT04733534) is evaluating the efficacy of senolytic regimens—the combination of dasatinib plus quercetin and fisetin alone—in improving frailty and reducing cellular senescence in adult survivors of childhood cancer. A positive outcome from this trial could establish a foundation for broader applications in managing frailty and vascular health in older adults, particularly those at risk for cardiovascular decline.

##### 2.4.3.2 Stem cell mobilization

Stem cell mobilization refers to the process of stimulating the release of EPCs from the bone marrow into the bloodstream, enabling them to migrate to sites of vascular injury and participate in repair. These cells are crucial for the maintenance of endothelial function and the regeneration of damaged blood vessels, making them essential for cardiovascular health. The cytokine granulocyte-macrophage colony-stimulating factor (GM-CSF) has been shown to effectively mobilize EPCs. For instance, studies demonstrate that low-dose GM-CSF mobilizes EPCs, accelerating reendothelialization after intravascular radiation. Similarly, in patients with peripheral artery disease, GM-CSF therapy mobilizes progenitor cells and improves endothelial function (117, 118).

##### 2.4.3.3 Angiogenic gene therapy

Angiogenic gene therapy involves the use of genetic material, such as VEGF, to stimulate the formation of new blood vessels, a process known as angiogenesis (119). This therapy aims to improve blood flow and tissue perfusion, particularly in areas with poor circulation, which is often a concern in individuals with conditions such as frailty and CVD. VEGF plays a pivotal role in promoting angiogenesis and enhancing endothelial function, both of which are crucial for vascular health and repair.

Recent studies have shown that VEGF gene therapy can significantly improve vascular perfusion and endothelial function. For example, in a study using a rat model of myocardial infarction (MI), an injectable alginate hydrogel loaded with AAV9-VEGF and conductive polyaniline nanorods enhanced angiogenesis, reduced oxidative stress, and restored cardiac function, demonstrating the potential of combined VEGF gene therapy and conductive biomaterials to promote heart repair after ischemic injury (120). Additionally, VEGF therapy has also been shown to mobilize EPCs, which play an essential role in repairing damaged blood vessels (119). These findings suggest that angiogenic gene therapy could provide a novel approach for treating frailty-related vascular decline and improving overall cardiovascular function in frail older adults. If validated in larger trials, this therapy could be an important tool for managing vascular dysfunction and frailty in individuals at high risk for cardiovascular events.

Collectively, these emerging therapies hold the potential to disrupt the cycle of vascular decline and frailty, ultimately improving clinical outcomes in frail older adults with CVD.

#### 2.4.4 Addressing the metabolic-frailty-CVD axis

The interplay between frailty and CVD is further complicated by mitochondrial dysfunction and oxidative stress, which impair energy metabolism in both skeletal muscle and cardiac tissue (40). These alterations contribute to cardiac remodeling, diastolic dysfunction, and a reduced physiological reserve, which are hallmarks of both frailty and CVD (39). Therapeutic strategies targeting adipose tissue inflammation (e.g., through weight loss, exercise, or anti-inflammatory agents) may help mitigate this vicious cycle, offering potential interventions to improve frailty-related cardiovascular decline (34).

Furthermore, neurohormonal imbalances, such as reduced levels of growth hormone, IGF-1, and sex steroids, play crucial roles in the deterioration of both muscle mass and cardiovascular resilience. Interventions targeting these axes, including ACE inhibitors, mineralocorticoid receptor antagonists, or cortisol-modulating therapies, may help modulate frailty-related CVD risk.

#### 2.4.5 The role of autonomic imbalance

Excessive sympathetic overdrive contributes to arrhythmias, myocardial ischemia, and cardiac remodeling, whereas blunted vagal activity worsens prognosis in conditions such as HF and ACS (121). This imbalance is associated with poor outcomes, including sudden cardiac death. Restoring autonomic balance through physical activity, biofeedback, beta-blockers, and parasympathetic stimulation techniques (e.g., vagus nerve stimulation) may offer dual benefits in reducing frailty and cardiovascular risk.

In summary, addressing frailty and CVD in older adults requires a multidisciplinary, individualized approach. While traditional risk tools often fail to account for frailty, emerging therapeutic strategies, such as senolytics, stem cell mobilization, and angiogenic gene therapy, offer hope for restoring vascular repair capacity and breaking the cycle of vascular decline. Additionally, targeting mitochondrial dysfunction, oxidative stress, adipose tissue inflammation, and neurohormonal imbalances provides a comprehensive approach for managing frailty-related cardiovascular decline.

### 2.5 Knowledge gaps and controversies

Despite significant advances in understanding the frailty-CVD relationship, several critical knowledge gaps and controversies persist that challenge both clinical practice and research. These unresolved issues span methodological approaches, the translation of basic science into therapies, and the fundamental interpretation of epidemiological data. This section examines three key areas of contention: the lack of standardization in frailty assessment, the premature enthusiasm for emerging biological therapies, and the inherent limitations of observational data in establishing causality.

#### 2.5.1 Assessment tool controversies

A central challenge is the lack of a gold standard frailty assessment, leading to a fundamental trade-off between practicality and comprehensiveness. Brief tools like the CFS enable rapid screening in busy clinical settings but may overlook key deficits. Conversely, detailed instruments like the FI offer greater precision but are often too resource-intensive for routine use. This discrepancy means that a single patient may be classified differently depending on the tool applied, directly impacting prevalence estimates and risk stratification. The optimal integration of these tools into specific cardiovascular care pathways—such as pre-procedural planning vs. primary care screening—remains an area of active debate and requires further standardization.

#### 2.5.2 Limitations of emerging therapies

While novel interventions like senolytics (e.g., dasatinib plus quercetin) and stem cell mobilizers (e.g., GM-CSF) show preclinical promise for targeting shared aging pathways, it is crucial to highlight their current evidence shortcomings. The existing data are primarily from small-scale, early-phase trials with short follow-up periods. Significant limitations include a lack of large randomized controlled trials demonstrating efficacy on hard cardiovascular outcomes, undefined long-term safety data in older, multimorbid populations, and unproven direct benefits on frailty status. Consequently, these therapies must be considered investigational and are not yet supported by robust evidence for clinical use outside of a trial setting.

#### 2.5.3 Methodological constraints and future directions

The prevailing evidence for the bidirectional frailty-CVD relationship stems largely from observational studies, which can establish association but cannot definitively prove causality. A key challenge is discerning whether frailty directly causes CVD progression, CVD causes frailty, or if both are parallel outcomes of shared underlying biological aging processes. Furthermore, most research aggregates “older adults” into broad categories, potentially masking important mechanistic differences between the young-old and oldest-old. Future studies should prioritize longitudinal designs and interventional trials to establish causality and elucidate how the frailty-CVD interplay varies across ages, sexes, and ethnicities, thereby enabling truly personalized care.

## 3 Conclusion

Frailty is increasingly recognized as a key factor influencing cardiovascular outcomes, particularly in older adults, through a bidirectional relationship where each condition worsens the other. This synergistic relationship leads to significantly poorer outcomes, yet frail patients often receive less intensive treatment. To better understand this link, future research should prioritize the exploration epigenetic mechanisms (e.g., DNA methylation), gut microbiota's role in inflammation and metabolic dysfunction, and biological targets like stem cell aging.

Clinically, the integration of standardized frailty assessments into cardiovascular care is urgently needed. This should be complemented by the development of biomarker-guided interventions, AI-driven monitoring, and the broader implementation of proven therapies such as exercise and nutritional support. From a policy perspective, integrating frailty into CVD guidelines and adopting multidisciplinary care models are essential to improve risk stratification and enable personalized treatment. Addressing frailty as a modifiable risk factor rather than an inevitable aspect of aging requires a multifaceted approach: advancing foundational research, refining clinical tools, and implementing supportive policy reforms. By prioritizing these strategies, healthcare systems can transform the management of frailty and significantly improve outcomes for the growing population of older adults with CVD.

## Glossary

ACE inhibitors: angiotensin-converting enzyme inhibitors
ACS: acute coronary syndrome
ADL: activities of daily living
ADMA: asymmetric dimethylarginine
AF: atrial fibrillation
AI: artificial intelligence
AMI: acute myocardial infarction
ANS: autonomic nervous system
ARBs: angiotensin receptor blockers
ATP: adenosine triphosphate
BNP: B-type natriuretic peptide
CABG: coronary artery bypass grafting
CAD: coronary artery disease
CAF: comprehensive assessment of frailty
CCI: Charlson Comorbidity Index
CDR: Cortisol-to-DHEA ratio
CFS: Clinical Frailty Scale
CI: confidence interval
CIEDs: cardiac implantable electronic devices
COH: consensus orthostatic hypotension
CRP: C-reactive protein
CSHA: Canadian Study of Health and Aging
CTR: cardiac telerehabilitation
CVD: cardiovascular disease
CXCR4: C-X-C Chemokine Receptor Type 4
DHEA: Dehydroepiandrosterone
DNA: deoxyribonucleic acid
Drp1: dynamin-related protein 1
EAT: epicardial adipose tissue
EC: endothelial cell
e-FI: electronic frailty index
EFT: Essential Frailty Toolset
EPCs: endothelial progenitor cells
ESC: European Society of Cardiology
e-SIF: Electronic Screening Instrument for Frailty
FI: Frailty Index
FP: Fried Phenotype
FTA: frailty trajectory analysis system
GM-CSF: granulocyte-macrophage colony-stimulating factor
GRACE: Global Registry of Acute Coronary Events
HF: heart failure
HFpEF: heart failure with preserved ejection fraction
HFrEF: heart failure with reduced ejection fraction
HIF-1*α*: hypoxia-inducible factor 1-alpha
HPA axis: Hypothalamic‒Pituitary‒Adrenal axis
HR: hazard ratio
IGF-1: Insulin-like Growth Factor-1
IL-1β: Interleukin-1 beta
IL-6: Interleukin-6
IOH: initial orthostatic hypotension
LDL: low-density lipoprotein
LIFE study: Lifestyle Interventions and Independence for Elders
MACE: major adverse cardiovascular events
MMSE: Mini-Mental State Examination
MNA: Mini Nutritional Assessment
mTOR: mechanistic target of rapamycin
MSSA: MacArthur Study of Successful Aging
mtDNA: mitochondrial DNA
NO: nitric oxide
NOS: nitric oxide synthase
NLRP3: NLR Family Pyrin Domain Containing 3
NT-proBNP: N-terminal pro-B-type natriuretic peptide
NSTE-ACS: Non-ST Elevation Acute Coronary Syndrome
OH: orthostatic hypotension
OXPHOS: oxidative phosphorylation
oxLDL: oxidized low-density lipoprotein
PCI: percutaneous coronary intervention
PGC-1*α*: peroxisome proliferator-activated receptor gamma coactivator 1-alpha
PVD: peripheral vascular disease
RAAS: renin–angiotensin–aldosterone system
ROS: reactive oxygen specie
SDF-1: stromal cell-derived factor-1
SPPB: Short Physical Performance Battery
SPRINT-T: Systematic Intervention for Frailty Prevention Trial
TAVR: transcatheter aortic valve replacement
TFAM: Mitochondrial Transcription Factor A
TFI: Tilburg Frailty Indicator
TLRs: Toll-like receptors
TNF-α: tumor necrosis factor-alpha
TUG: Timed Up and Go test
VEGF: Vascular Endothelial Growth Factor
VHD: valvular heart disease
VIVIFRAIL: multicomponent exercise program for frailty
VSMC: vascular smooth muscle cell
